# A Theory-Informed Review of Food Insecurity in Higher Education: Determinants, Gaps, and Policy Implications

**DOI:** 10.1016/j.advnut.2025.100462

**Published:** 2025-06-12

**Authors:** Hiwot Abebe Haileslassie

**Affiliations:** Agricultural, Food and Nutritional Science, University of Alberta, Edmonton, Alberta, Canada

Students pursue higher education to nourish their intellectual hunger and expand their knowledge. Yet, while striving to fulfill these goals, many endure hunger in a literal sense. This issue is part of a broader, systemic problem: food insecurity. Although not synonymous, hunger and food insecurity are closely linked, with the latter often leading to the former. A growing body of evidence reveals that students are experiencing food insecurity at an alarmingly high rate. The USDA defines food insecurity as “limited or uncertain availability of nutritionally adequate and safe foods, or limited or uncertain ability to acquire acceptable foods in socially acceptable ways” [1]. This definition does not directly measure hunger itself but instead reflects the economic and social conditions that may lead to hunger [2].

The consequences of food insecurity are wide-ranging and severe. Students experiencing food insecurity tend to have poor mental and physical health, diminished academic performance, and higher dropout rates. A recent study by Brownfield et al. [3] highlighted the direct impact of food insecurity on students, noting a clear correlation between food insecurity and lower academic achievement as measured by grades. Addressing this issue not only supports individual well-being and educational success but also aligns with global initiatives such as Sustainable Development Goal 2, which aims to end hunger in all its forms by 2030. In a recent issue of *Advances in Nutrition*, Sklar et al. [4] offered a comprehensive scoping review of 53 studies published in the past decade that investigated food insecurity in higher education through the lens of Social Cognitive Theory (SCT).

## The SCT Framework: A Valuable Lens

Sklar et al. [4] provided a structured synthesis of current scientific evidence while identifying key gaps and strategic pathways forward. The distinct strength of this review lies in its theoretical orientation employing SCT, which emphasizes the interaction of personal, behavioral, and environmental influences as a framework. This reciprocal determinism [5] approach allows a more nuanced understanding of why students experience food insecurity and how their experiences are shaped by intersecting factors such as knowledge, attitude, food access, resource usage, barriers and stigma, practices, skills, and cooking self-efficacy.

## Theory-Informed Determinants of Food Insecurity

Interventions grounded in behavior change theories are generally more effective and allow researchers to pinpoint the key factors driving behavior change. Sklar et al. [4] classified the determinants of food insecurity into 3 primary domains: Personal/Cognitive Factors, Behavioral Factors, and Environmental Factors. Most of the reviewed studies found that nutrition, food literacy, or budgeting knowledge was associated with improved food security, healthier dietary patterns, and positive behaviors such as informed food purchasing and meal preparation. Food-related skills and cooking self-efficacy were found to be positively associated with food security, with intervention-based programs improving cooking frequency, diet quality, and confidence in meal preparation. However, some critics may argue that focusing primarily on individual knowledge and behavior risks overlooking broader structural determinants of food insecurity, such as rising tuition costs, inadequate financial aid, and the high cost of living, which are beyond the control of individual students. Moreover, knowledge alone does not guarantee action; factors such as time constraints, cultural relevance of food options, and mental health challenges may limit the application of food literacy skills. Efforts to build skills and enhance knowledge are valuable; however, because food insecurity is multifaceted, these efforts must be paired with institutional and policy-level interventions to create meaningful and sustainable change.

Studies consistently reveal that college students often perceive themselves as more food secure than they actually are, leading to underutilization of available food resources. Students tended to normalize food insecurity by viewing it as an expected and inevitable part of the college experience. Research across 39 studies highlights the impact of this normalization; food-insecure students frequently rely on coping strategies such as skipping meals, consuming low-cost processed foods, and making financial trade-offs, often at the expense of nutrition and health [4]. Despite the persistent stigma, lack of awareness, and barriers such as time, transportation, and eligibility confusion, studies demonstrate that students who use food access resources, such as on-campus pantries, significantly lower rates of food insecurity. It should be noted that although helpful, they serve only as temporary relief and fail to address the root causes of food insecurity.

## Gaps in Literature

Although the public health significance of student food insecurity is well established, key research gaps remain, including limited comprehensive studies that capture all components of SCT and an overreliance on cross-sectional data. Sklar et al. [4] emphasize that longitudinal studies are essential for understanding temporal variations in food insecurity and designing timely and adaptive interventions. These recommendations align with the priority research gaps identified by Landry et al. [6]. Recognizing the widespread food insecurity among students, discussions have focused on revising policy to improve eligibility criteria for food assistance programs such as for postsecondary students and on enhancing student awareness of these resources [7]. The lack of a validated questionnaire for assessing food insecurity in higher education was evident in the review [4]. Nikolaus et al. [8] also argue the current tools may not fully reflect the unique challenges faced by higher education students.

## Global Relevance and Broader Implications

Although Sklar et al.’s [4] review focuses on studies in the United States, similar patterns have emerged globally [9]. Food insecurity in higher education is a pressing and multifaceted challenge. It undermines student well-being, academic success, and social equity. The SCT provides a robust lens for understanding this issue, highlighting the complex interplay of personal, behavioral, and environmental factors. Moving forward, a balanced approach that combines individual skill-building with institutional reform and policy change is essential. The goal of higher education should be not only to provide intellectual nourishment but also to ensure that all students have the opportunity to thrive.

## Funding

The author reported no funding received for this work.

## Conflict of interest

The author reports no conflict of interest.
